# Molecular ordering at electrified interfaces: Template and potential effects

**DOI:** 10.3762/bjoc.10.233

**Published:** 2014-09-23

**Authors:** Thanh Hai Phan, Klaus Wandelt

**Affiliations:** 1 Institute of Physical and Theoretical Chemistry, University of Bonn, Wegelerstr. 12, 53115 Bonn, Germany; 2 Laboratory of Photochemistry and Spectroscopy, Department of Chemistry, Catholic University of Leuven, Celestijnenlaan 200F, B-3001, Hevelee, Belgium; 3 Physics Department, Quynhon University, 170 An Duong Vuong; Quynhon, Vietnam; 4 Institute of Experimental Physics, University of Wroclaw, MaxaBorna 9, 50-204, Wroclaw, Poland

**Keywords:** cyclic voltammogram, scanning tunneling microscopy, self-assembly, template effect, viologen

## Abstract

A combination of cyclic voltammetry and in situ scanning tunneling microscopy was employed to examine the adsorption and phase transition of 1,1’-dibenzyl-4,4’-bipyridinium molecules (abbreviated as DBV^2+^) on a chloride-modified Cu(111) electrode surface. The cyclic voltammogram (CV) of the Cu(111) electrode exposed to a mixture of 10 mM HCl and 0.1 mM DBVCl_2_ shows three distinguishable pairs of current waves P1/P’1, P2/P’2, and P3/P’3 which are assigned to two reversible electron transfer steps, representing the reduction of the dicationic DBV^2+^ to the corresponding radical monocationic DBV^+•^ (P1/P’1) and then to the uncharged DBV^0^ (P3/P’3) species, respectively, as well as the chloride desorption/readsorption processes (P2/P’2). At positive potentials (i.e., above P1) the DBV^2+^ molecules spontaneously adsorb and form a highly ordered phase on the c(p × √3)-precovered Cl/Cu(111) electrode surface. A key element of this DBV^2+^ adlayer is an assembly of two individual DBV^2+^ species which, lined up, forms a so-called “herring-bone” structure. Upon lowering the electrode potential the first electron transfer step (at P1) causes a phase transition from the DBV^2+^-related herring-bone phase to the so-called "alternating stripe" pattern built up by the DBV^+•^ species following a nucleation and growth mechanism. Comparison of both observed structures with those found earlier at different electrode potentials on a c(2 × 2)Cl-precovered Cu(100) electrode surface enables a clear assessment of the relative importance of adsorbate–substrate and adsorbate–adsorbate interactions, i.e., template vs self-assembly effects, in the structure formation process of DBV cations on these modified Cu electrode surfaces.

## Introduction

The precise control of the self-organization of molecular layers on either conducting or dielectric substrates is regarded as one of the key factors in the successful design, characterization and fabrication of nanoscale molecular devices [1–4]. A big challenge for surface scientists is, thus, to find suitable model systems which enable to investigate the driving forces of molecular self-organization on surfaces and to simulate the working principles of the derived molecular devices. This so-called “bottom-up” strategy, i.e., the formation of supramolecular structures from vapor deposited simpler building blocks has become an important research direction in ultra-high vacuum (UHV) based surface science in recent years. However, promising organic compounds may not remain intact volatile, and may thus not be deposited via the gas phase. In those cases, it may be a promising strategy to deposit these molecules from solution instead. Besides, this preparation route is probably also more economic than operating a vacuum evaporation system, in particular if the organic building blocks come in water-soluble form.

In principle, the self-organization process of molecular adsorbates is driven by the interplay between adsorbate–adsorbate and adsorbate–substrate interactions. While the former depend on the specific nature of the molecular building blocks, e.g., their shape, polarity, functional groups, etc., and may include all possible interactions from van der Waals forces to covalent bonds, the latter are described by the so-called “corrugation function”, i.e., the two-dimensional (2D) potential energy landscape representing the minimum total energy for all possible adsorbate–substrate configurations and, thereby, the interaction strength between the substrate and an adsorbate molecule at any surface site. If, in equilibrium, the adsorbate–adsorbate interactions dominate over the adsorbate–substrate interactions the molecules will essentially “self-assemble” independent of the substrate surface. If, however, the adsorbate–substrate interactions are very strong compared to the intermolecular forces the substrate will influence the structure of the adsorbate layer, provided a sufficient surface mobility allows the adsorbed molecules to reach their equilibrium positions, i.e., minima in the corrugation function. In this case the substrate surface acts as a “template”. Under UHV conditions the activation energy for structural equilibration is usually provided by heating the substrate. If however, the organic species are deposited in ionic form from aqueous solution, as done in the present work, the obtained structure will additionally be influenced by electrostatic forces acting between the molecules and the substrate as well as between the molecules themselves. In this respect electrochemical deposition has the additional advantage that these electrostatic interactions can be “tuned” by the electrochemical potential in two ways. On the one hand the mere charge density at the electrode surface itself determines the electrostatic forces between adsorbed ions, not only the organic species but also possibly co-adsorbed other ions present in the solution, and the substrate. On the other hand, driven by the electrode potential the molecular ions may undergo redox-reactions, thereby changing their own charge state. Both cases are expected to influence the deposition and structure formation of the molecular layers.

In this paper we will present results on the self-organization of 1,1’-dibenzyl-4,4’-bipyridinium, in short dibenzyl-viologen (DBV), cations on a chloride precovered Cu(111) electrode surface. A comparison of these findings with those described earlier for the same molecules on a chloride-modified Cu(100) electrode [5–7], will then enable us to arrive at a generalized picture of the influence of template and potential effects on the structure formation of these molecular ions on both chloride modified copper single crystal surfaces of different symmetry.

The motivation for the choice of viologen molecules is twofold. On the one hand molecular viologen-based self-assemblies have attracted a great deal of attention in recent years due to their widespread applications in electronic devices [8–9], and light-harvesting operators [10]. On the other hand the electrochemistry of viologens in solution is well documented in the literature [11–12]. In dicationic form dibenzyl-viologen molecules (DBV^2+^) are well-known to undergo two successive reversible electron transfer steps yielding first the corresponding monocation radical DBV^+•^ and then the uncharged viologen species DBV^0^, respectively. The first investigations on the surface redox chemistry as well as the self-assembly of DBV-species on a chloride modified Cu(100) surface, were presented by Safarowsky et al. and Pham et al. [5–7]. In the present paper we will describe for the first time the structural properties of self-assembled DBV on a chloride terminated Cu(111) electrode surface. Their comparison with the previous results obtained on the chloride precovered Cu(100) surface will clearly demonstrate the relative importance of adsorbate–substrate and adsorbate–adsorbate interactions, i.e., template-effects vs self-assembly, at different electrode potentials.

## Results

### Electrochemical characterization

**Figure 1:**
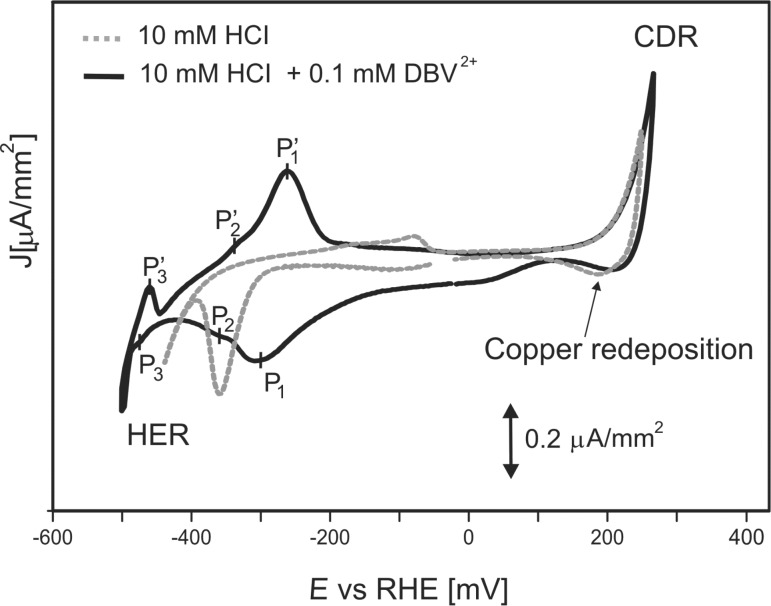
Cyclic voltammograms of Cu(111) in pure 10 mM HCl (dashed grey curve) and in viologen molecules containing (10mM HCl + 0.1 mM DBV^2+^) solution (solid black curve); d*E*/d*t* = 10 mV/s; *E* = electrode potenial. CDR = copper dissolution reaction, HER = hydrogen evolution reaction. Reproduced with the permission from [16].

The electrochemical characterization of the Cu(111) surface in both pure 10 mM HCl and the viologen containing (10 mM HCl + 0.1 mM DBV^2^**^+^**) solution was done using cyclic voltammetry. Representative steady-state CVs are shown in Figure 1. The potential window of the Cu(111) electrode in the pure supporting electrolyte (10 mM HCl) is limited by the oxidative copper dissolution reaction (CDR) at the anodic limit and the reductive hydrogen evolution reaction (HER) at the cathodic limit. At intermediate potentials, two peaks are seen which are due to chloride desorption/adsorption at −360 mV and −80 mV, respectively. Compared to this CV in pure hydrochloric acid, drastic changes are found in the cyclic voltamogram of the Cu(111) electrode in contact with the electrolyte containing 0.1 mM DBV^2+^.

The first difference relates to a considerable shift of the HER towards lower potentials in the presence of the organic overlayer. This shift is most likely caused by viologen molecules blocking the most reactive surface sites for this reaction. The same effect was also reported by Pham et al. [6] and Safarowsky et al. [7] using a Cu(100) crystal as working electrode.

**Figure 2:**
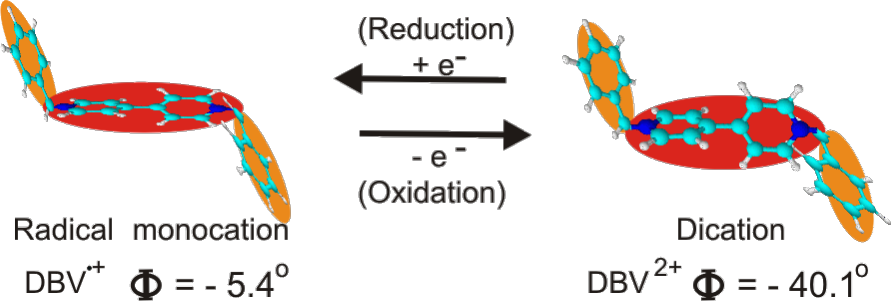
Reversible redox-state of viologen molecule; Φ = dihedral angle of the respective bipyridinium core.

The second most obvious deviation concerns the appearance of three new pairs of peaks at potentials close to the HER. These additional current waves, namely P_1_/P_1_’ and P_2_/P_2_’ and P_3_/P_3_’, are assigned to viologen-related redox-processes (P_1_/P_1_’ and P_3_/P_3_’) [5–7,13–16], as well as to an order/disorder phase transition due to chloride desorption/adsorption [17]. As mentioned in the Introduction, the viologen dication (DBV^2+^) is known to undergo two successive one-electron transfer steps in the electrochemical environment forming first the viologen monocation radical (DBV^+•^) (Figure 2) and then the uncharged molecule (DBV^0^) (for details see [5–6,13–16], and the papers cited therein). While the dication and monocation radicals are soluble in aqueous solutions, the uncharged molecules can accumulate at the electrode surface due to their hydrophobic properties [4].

The actual shape of the black cyclic voltammogram in Figure 1, in particular the relative intensities of the various peaks can be understood when considering the involvement of “solution species” and “surface limited” reactions, respectively. Starting at positive potentials, i.e., above P_1_/P_1_', the reduction/re-oxidation of both, the limited number of pre-adsorbed viologen dications (see XPS evidence below) and the continuously arriving viologen cations from solution, can be described by

DBV^2+^ + e^−^ 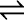 DBV^+•^

While DBV^+•^ species leaving the surface are known to form dimers in solution [5], DBV^+^**^•^** species staying on the surface may also form polymeric chains (see below):

2 DBV^+•^ 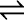 [DBV_2_]^2+^ (solution)

*n* DBV^+•^ 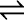 [DBV*_n_*]*^n^*^+^ (surface)

The reduction of the surface-confined species may even occur at an electrode potential different form that for the reduction of “solution species”.

The further reduction of the monocation radicals to the fully uncharged viologen molecule DBV^0^ (peak P_3_ in Figure 1)

*n* DBV^+•^+ *n* e^−^ 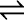 *n* DBV^0^

occurs already within the regime of massive hydrogen evolution. Since under these conditions reliable in situ STM measurements are not possible, the influence of this second reduction step on the structure of the deposit is not considered further here.

In the following sections we will now present and discuss in situ STM images as obtained for the electrode surface in different potential regimes. We start at potentials where the molecules retain their dicationic character (DBV^2+^) in the adsorbed state, and then continue with results taken at potentials where the adsorbed molecules have undergone the first one-electron reduction step (at P_1_ in Figure 1) and exist in their monocation radical form DBV^+•^. These images clearly show the decisive influence of the respective charge state of the molecular species on the structure of the adsorbed DBV layer.

### Structural characterizations

**Figure 3:**
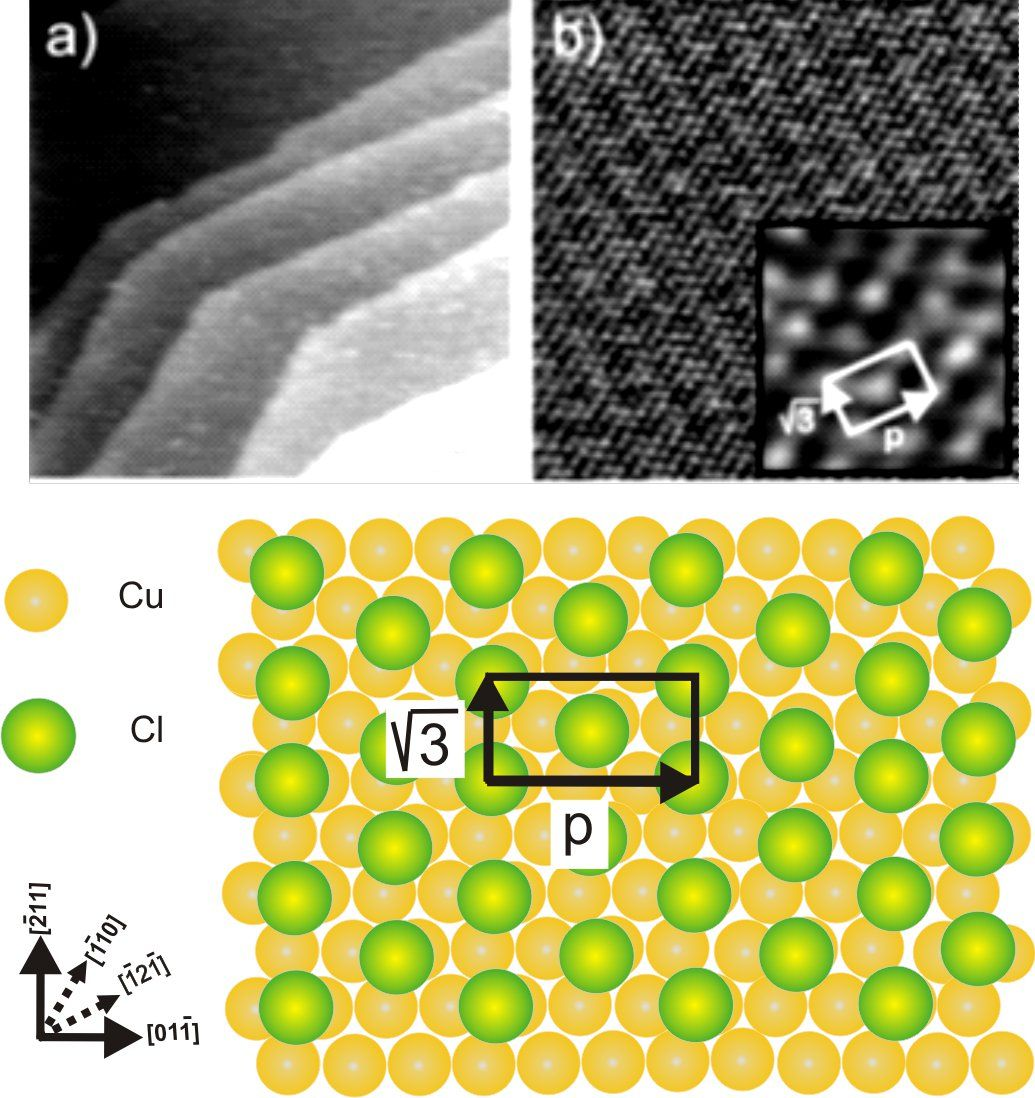
Chloride-modified Cu(111) surface : a) STM image 70 nm × 70 nm, bias voltage *U*_b_ +220 mV, tunneling current *I*_t_ = 0.2 nA, *E* = 50 mV; b) STM image of the c(p × √3)Cl structure: 14.4 nm × 14.4 nm, 5.58 nm × 5.58 nm, *I*_t_ = 2.0 nA, *U*_b_ = 75 mV, *E* = 0.0 mV; c) Hard-sphere model of the chloride-modified Cu(111) surface.

As documented in the literature [18–22] a well-ordered c(p × √3) layer of adsorbed chloride anions is formed on the Cu(111) surface (Figure 3) in the supporting HCl electrolyte, which, starting from a hexagonal c(√3 × √3)R30° structure at negative potentials exhibits the phenomenon of reversible electrocompression into a uniaxially incommensurate c(p × √3) structure with rectangular unit cell at positive potentials. This c(p × √3)Cl structure remains stable in the potential range between the copper dissolution reaction and about −300 mV (see Figure 1). In contrast to the larger halides, e.g., bromide and iodide, the chloride anions retain to a large extent their negative charge upon adsorption [23–24]. Hence, this regular array of anions can be regarded, similar to the c(2 × 2)Cl layer on Cu(100) (see below), as a suitable template for the adsorption of positively charged organic molecules.

**Figure 4:**
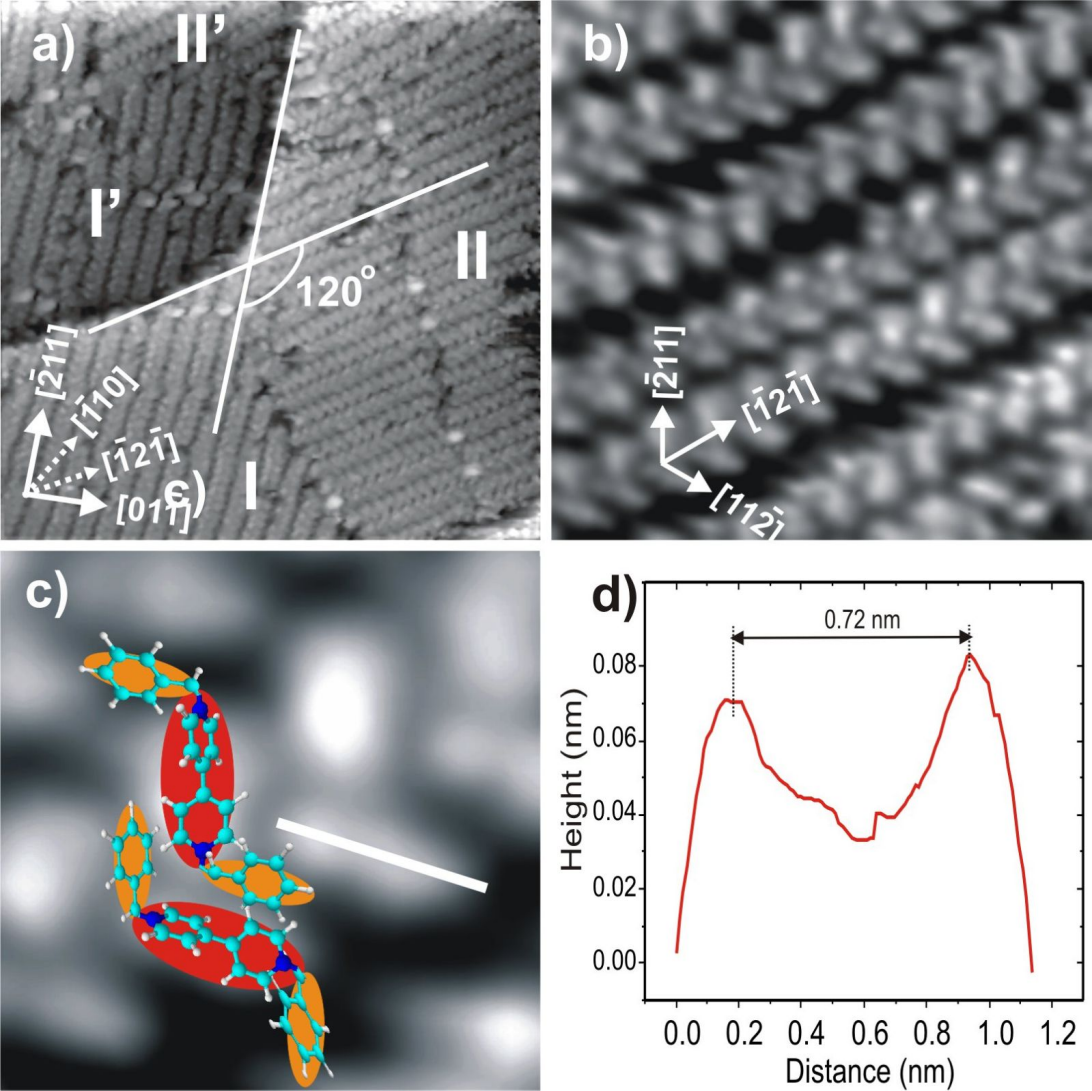
Typical STM images of the surface morphology and high-resolution images of the DBV^2+^ related herring-bone phase on Cl/Cu(111): a) Surface morphology of the surface with a characteristic substrate step angle of 120° and two existing rotational domains (I/II; I'/II') of the DBV^2+^ herring-bone phase: 46.67 nm × 46.67 nm, *U*_b_ = 141 mV, *I*_t_ = 0.3 nA, *E* = −180 mV; b,c) Medium-scale (20.73 nm × 20.73 nm) and high-resolution (2.8 nm × 2.8 nm) STM image of the herring-bone phase, the latter showing two individual DBV^2+^ molecules in each structural element: *U*_b_ = 386 mV, *I*_t_ = 0.1 nA, *E* = +10 mV; d) Line profile recorded along the white line in Figure 4c indicating the length of the dipyridinium group of about 0.72 nm in perfect agreement with the N–N distance within the DBV^2+^ molecule. Figure 4a is reproduced with permission from [16].

Exposing the c(p × √3)Cl terminated Cu(111) surface to the electrolyte containing DBV^2+^ ions at potentials between −50 mV and +50 mV vs RHE, i.e., in the potential regime above the first reduction peak P1 in Figure 1, results in the instantaneous formation of a highly ordered DBV^2+^ film. Figure 4 shows representative STM images describing the surface morphology and molecular structure of the DBV^2+^adlayer. First, the straight step-edges in Figure 4a running by each other at a typical angle of 120° still remain, providing a first indication of the persistence of the c(p × √3)Cl layer underneath the organic molecules (see also Figure 6). This indicates that the DBV^2+^ adlayer has no significant impact on the substrate-surface morphology, which is governed by the chemisorptive Cu–Cl bond. Two distinguishable domains rotated by an angle of 120° with respect to each other, denoted as I/I’ and II/II’ on the two different terraces shown, are observed in Figure 4a. A close inspection of the molecular arrangement makes it clear that the DBV^2+^ molecular rows within the domains are oriented parallel to step directions, the latter being aligned along the close-packed anion rows within the c(p × √3) chloride structure underneath [17,25]. As a result, the DBV^2+^ molecular rows are oriented parallel to the commensurate direction of the chloride lattice. Alternatively, they are aligned parallel to the 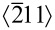 directions of the Cu(111) substrate (see Figure 3).

On the molecular level the rows consist of units of two bright oval dots assigned to the bipyridinium cores of individual DBV^2+^ molecules (Figure 2) that meet each other by an angle of 120 ± 2°. Using a line profile measurement along the white line in Figure 4c gives a length of about 0.72 ± 0.01 nm for one of the units (Figure 4d). This value is in complete agreement with the N–N distance of 0.71 nm within the DBV^2+^ molecules [5,12]. Based on this agreement in size, the given angle of 120°, and in particular, the consideration of electrostatic interactions between the bipyridinium core and the benzyl groups (for more details see the discussion below) we propose the molecular arrangement as shown in Figure 4c.

**Figure 5:**
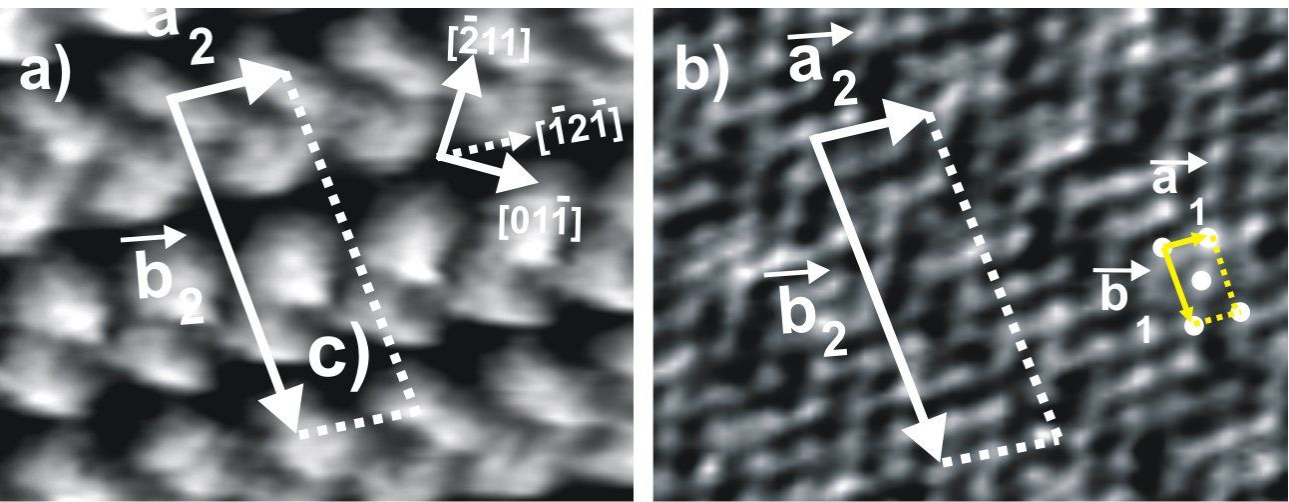
Structural correlation between the ordered DBV^2+^ herring-bone phase and the anionic chloride lattice underneath: a) 6.5 nm × 4.1 nm, *U*_b_ = 220 mV, *I*_t_ = 0.1 nA, *E* = −10 mV; b) 6.5 nm × 4.1 nm, *U*_b_ = 30 mV, *I*_t_ = 5.0 nA, *E* = −10 mV. 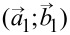 and 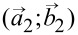 are the unit vectors of the chloride lattice and the herring-bone phase, respectively.

The structural correlation between the DBV^2+^ adlayer and the underlying chloride lattice could also be obtained by carefully varying the tunneling conditions [5,7,26]. Under “soft tunneling conditions”, i.e., with high bias voltage and low tunneling current, the characteristic features of the DBV^2+^ adlayer are observed (Figure 5a). Conversely, the chloride lattice underneath becomes visible when “drastic tunneling conditions” are applied, i.e., low bias voltage and high tunneling current. In this circumstance, the tunneling tip serves as a molecular brush to locally remove the DBV^2+^ overlayer, leaving the c(p × √3)-Cl lattice behind (Figure 5b). By comparing panels 5a and 5b, it becomes evident that the individual bipyridinium cores of the DBV^2+^ molecules are aligned parallel to the underlying close-packed chloride rows, i.e., parallel to the 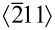 directions of the Cu(111) substrate, indicating the orienting effect of the lattice of the specifically adsorbed chloride anions on the structure of the adsorbed DBV^2+^ overlayer. This hints to a template effect rather than a mere self-assembly of the molecular dications on the Cl/Cu(111) surface. Again, based on a superposition of panels 5a and 5b, a precise determination of the DBV^2+^ unit cell with respect to the c(p × √3)-Cl phase underneath, the latter serving as an internal calibration lattice, is possible. As a result, the unit cell containing four DBV^2+^ molecules can be described by a rectangular (2 × 2p) mesh with respect to the c(p × √3)-Cl lattice (Figure 5a). The lattice constants are estimated to 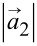 = 0.83 nm and 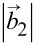 = 3.32 nm, respectively, enclosing an angle of 88 ± 2°. Alternatively, the unit-cell of the DBV^2+^ adlayer can be directly related to the copper substrate (1 × 1) mesh assuming a (4p × 2√3) coincidence mesh. The surface coverage per domain was also calculated as Θ = 0.25 ML with respect to the underlying chloride lattice, or 14.49·10^13^ molecules/cm^2^. A more detailed discussion of the molecular arrangement within the herringbone rows is postponed until section Discussion where we will also make a comparison with the corresponding system DBV on Cl/Cu(100).

**Figure 6:**
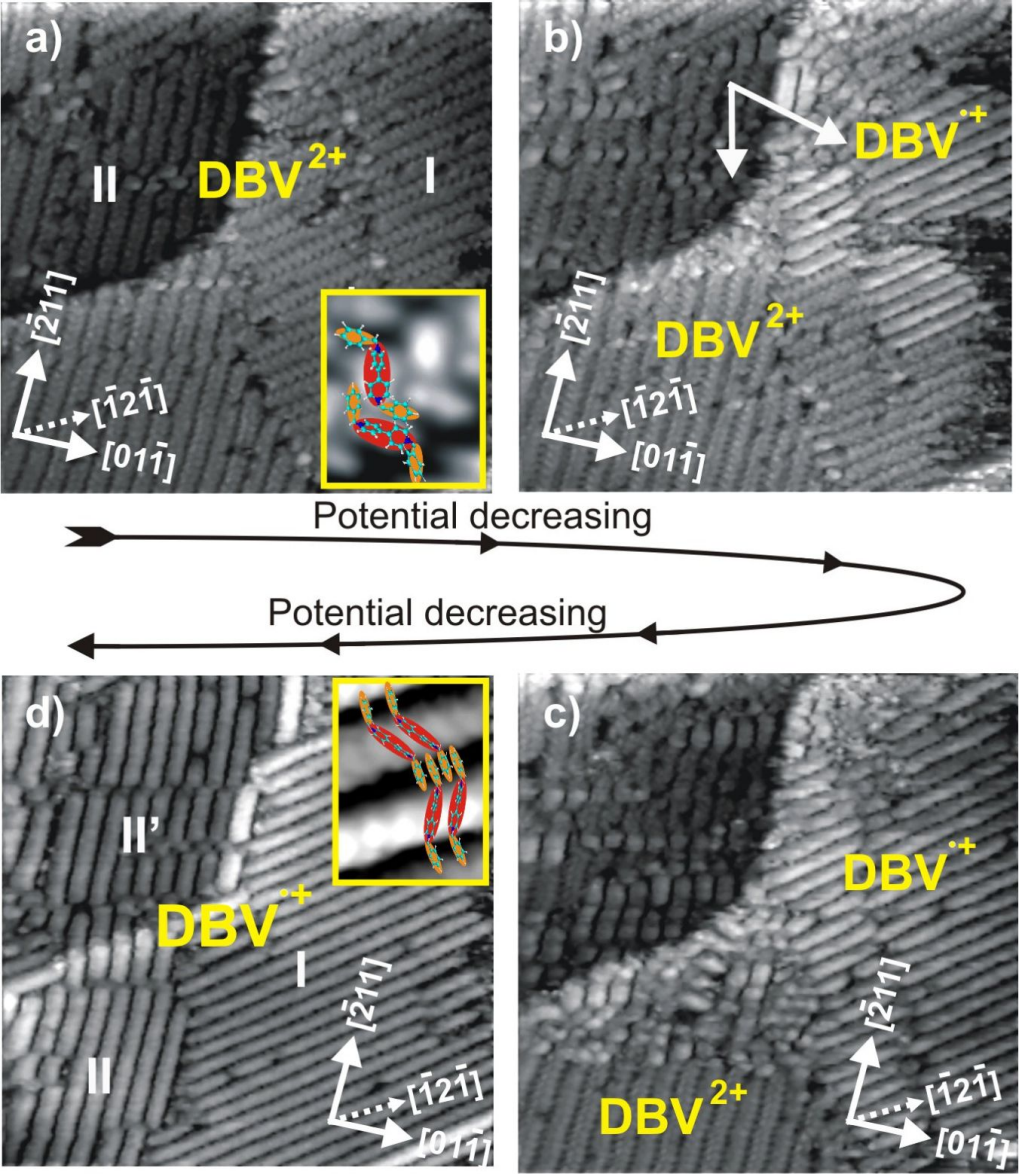
A series of STM images recorded with the same tunneling parameters (46.67 nm × 46.67 nm, *U*_b_ = 386 mV, *I*_t_ = 0.1 nA) showing the structural phase transition from the herring-bone phase to an alternating stacked stripe phase. Disintegration of the herring-bone phase and growth of the stripes starts preferentially at defect sites and step edges: a) *E* = −220 mV; b) *E* = −260 mV; c) *E* = −270 mV; d) *E* = −285 mV.

The DBV-dication based herring-bone structure remains stable in the potential range more positive than −240 mV vs RHE, but it decays below this potential, giving rise to a surface phase transition. Namely, the herring-bone phase disintegrates gradually when the electrode potential approaches peak P_1_ (cathodic potential sweep) in the solid black CV in Figure 1, where the viologen dication species (DBV^2+^) are reduced to the corresponding monocation radicals (DBV^+•^). Figure 6 shows this decay of the herring-bone structure and the simultaneous growth of the new stripe phase (Figure 6b and c) within the potential regime from *E* = −240 mV to *E* = −285 mV, in which the 120° step edge serves as a positional marker. The phase-transition process starts preferentially at point defects and domain boundaries (as marked by the white arrows in Figure 6b) because this requires a relatively low activation energy. Finally, the new stripe pattern is completed right after the potential reaches the value of *E* = −285 mV (Figure 6d). The observation of two rotational domains I and II (and I’ on the lower terrace, respectively) rotated by 120° elucidates the influence of the underlying substrate on the adsorption not only of the viologen dication (see above) but also of the monocation radical species. In fact, taking the symmetry of the substrate into account, three rotational domains in total should coexist on the Cu(111) surface. Additionally, the characteristic angle of 120° between substrate steps remains unaffected by this phase-transition process and rules out chloride desorption at these potentials. This rather hints to the molecular reduction as being the origin of the phase transition.

**Figure 7:**
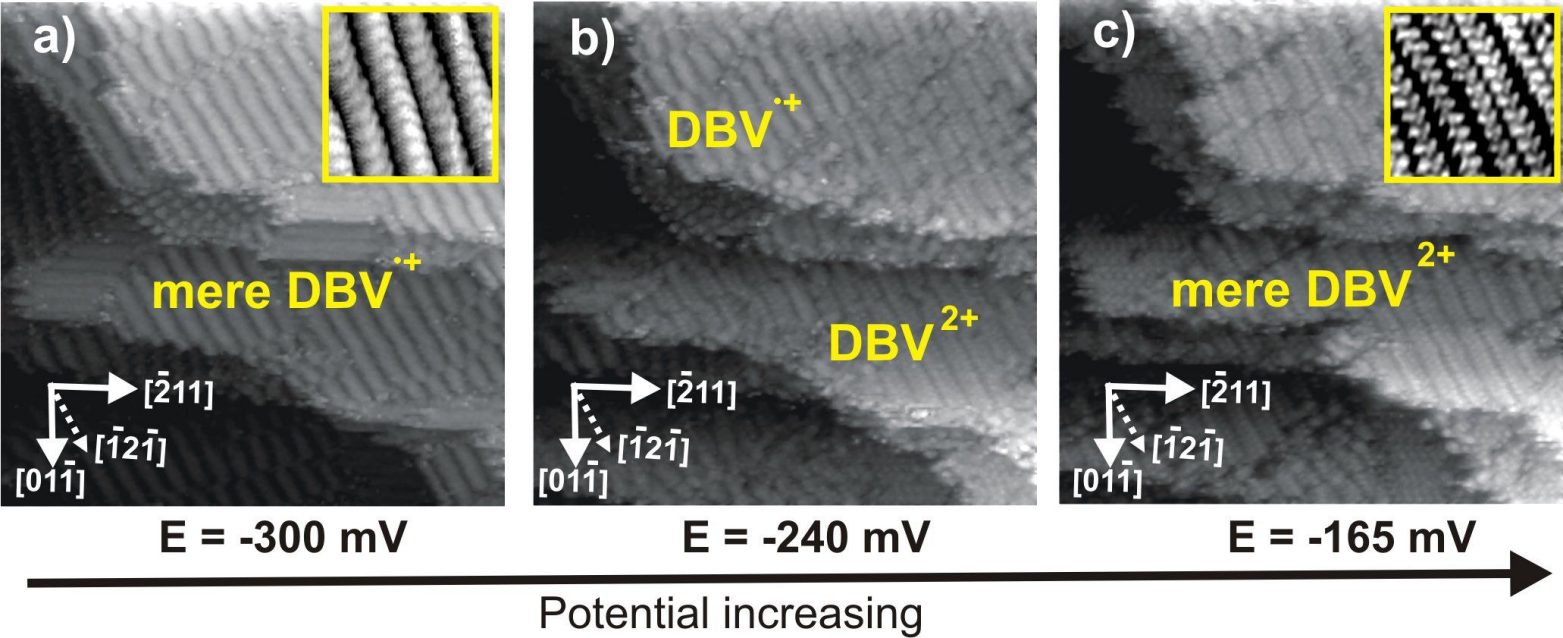
Series of STM images showing the desintegration of the stripe pattern and restoration of the corresponding herring-bone phase upon sweeping back toward positive potentials, 46.67 nm × 46.67 nm, *I*_t_ = 0.1 nA; a) *U*_b_ = 240 mV, *E* = −300 mV; b) *U*_b_ = 200 mV, *E* = −240 mV; b) *U*_b_ = 233 mV, *E* = −165 mV.

The first electron transfer-induced phase transition from the DBV^2+^ herring-bone phase to the stripe phase of the DBV^+•^ monocation radicals is a quasi-reversible process. The DBV^2+^ related herring-bone phase is gradually restored when the working potential is swept back towards the positive potential regime. A series of STM images recorded on the same surface area but at increasing electrode potentials, showing a phase transition from the stripe pattern back to the herring-bone phase, is shown in Figure 7. The stripe phase, as observed in Figure 7a (see inset), gradually desintegrates resulting in the reappearance of the herring-bone phase (Figure 7b), which finally completely replaces the stripe phase, as seen in Figure 7c (see inset). Similar to the transformation from the DBV^2+^ herring-bone phase to the DBV^+•^ stripe pattern, step-edges and defect points act as starting points for this phase-transition process. Comparing panels 7a and 7c, it becomes evident that the molecular row directions in the DBV^2+^ herring-bone phase are the same as in the DBV^+•^ stripe pattern, i.e., aligned parallel to substrate step edges. This observation again affirms the dominant role of interactions between the molecular adlayer and the underlying chloride lattice for the lateral ordering.

**Figure 8:**
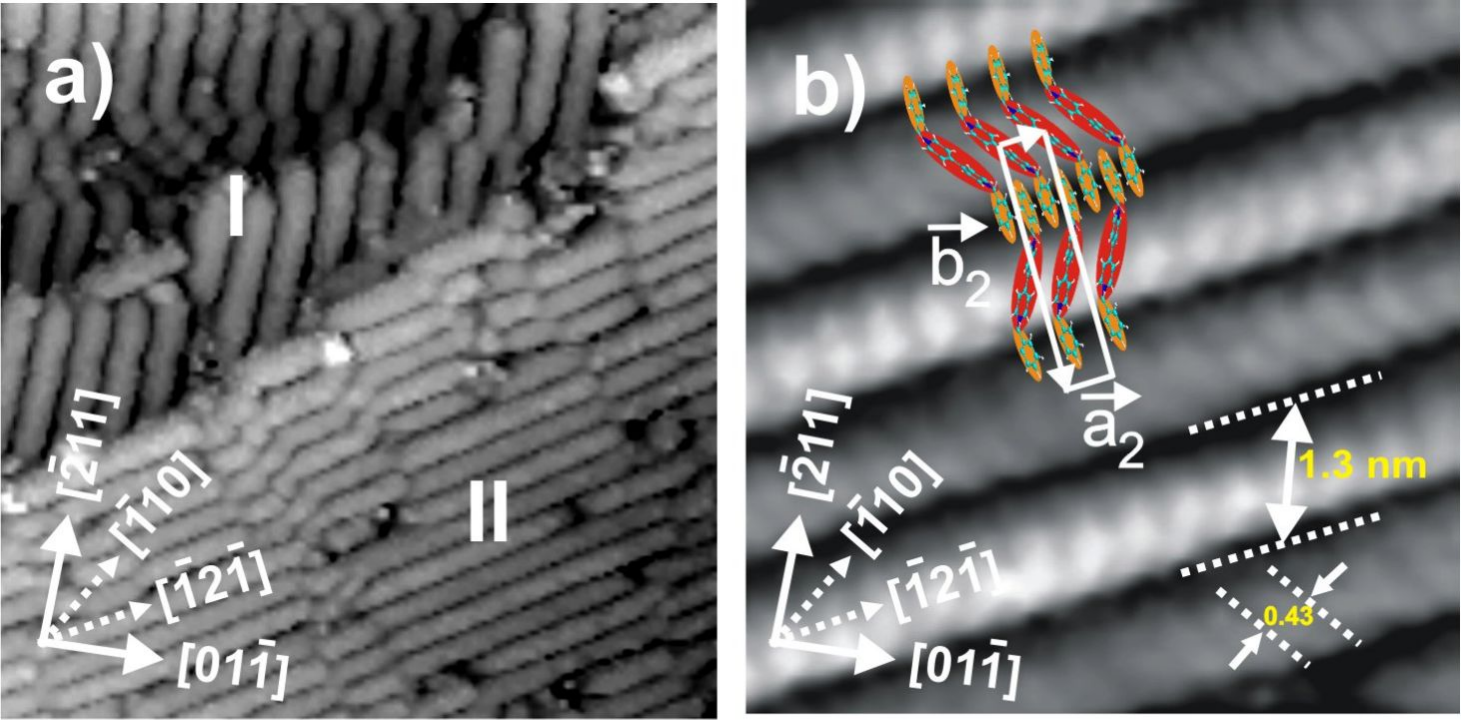
a) Typical STM image showing two rotational domains of the DBV^+•^ alterating stripe pattern (see text): 35.49 nm × 35.49 nm, *U*_b_ = 386 mV, *I*_t_ = 0.1 nA, *E*_w_ = −280 mV; b) Molecularly resolved STM image of the DBV^+•^ alterating stripe pattern, 5.06 nm × 5.06 nm, *U*_b_ = 298 mV, *I*_t_ = 0.1 nA, *E*_w_ = −286 mV. Reproduced with permission from [16].

Recalling the stripe phase of the DBV^+•^ molecules, Figure 8 presents typical meso- and molecular-scale STM images of this phase formed on the chloride-terminated Cu(111) electrode surface. As mentioned above and shown in Figure 8a the stripes are aligned parallel to the directions of step edges; these directions coincide with the close-packed chloride rows underneath, and, hence, the 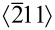 directions of the Cu(111) substrate (see [14] and papers cited therein). The higher resolution STM image in Figure 8b reveals further details of the internal structure of the rows, i.e., the molecular orientation and packing arrangement. The elongated and parallel discs within the rows are assigned to individual DBV^+•^ monocation radicals. Within one row all molecules have the same orientation, whereas the orientation of the monocation radicals in adjacent rows is alternating, in that the molecules in neighboring rows are rotated by 120° with respect to each other (redish discs in Figure 8b) leading to a zig-zag appearance. Hitherto this stripe pattern will therefore be termed "alternating stripe" pattern, in contrast to the findings on Cl/Cu(100) (see below). As a consequence not only the directions of the rows are aligned to a symmetry direction of the substrate surface, but also the bipyridinium cores are oriented in the direction of close-packed chloride rows underneath. Moreover, an even closer look reveals i) that within one row all molecules are imaged with the same intensity, suggesting equivalent adsorption sites, while ii) every second row of the "alternating stripe" structure appears slightly brighter in the STM image. Considering the uniaxial incommensuracy of the chloride structure underneath in the direction perpendicular to the rows (see Figure 3), the most likely explanation for the latter phenomenon is that molecules in adjacent rows are situated on non-equivalent chloride rows underneath. The intermolecular distances within one and the same row as well as between adjacent stripes are measured as *d*_as_= 0.43 ± 0.01 nm and *s*_as_= 1.3 ± 0.1 nm, respectively [16].

Applying different tunneling conditions, as mentioned above, the structural correlation between the DBV^+•^ adlayer and the underlying chloride lattice can again be made visible (not shown here). On the basis of a superposition of both lattices, the derived unit cell containing two DBV^+•^ molecules can be expressed by a (1 × 4) coincidence mesh with respect to the c(p × √3)Cl lattice with the lattice constants of 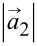 = 0.43 ± 0.01 nm and 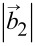 = 3.32 ± 0.1 nm, respectively. From this a DBV^+•^surface coverage per domain is calculated as Θ = 0.25 ML with respect to the c(p × √3)Cl layer serving as the template, or 14.30·10^13^ molecules/cm^2^.

## Discussion

**Figure 9:**
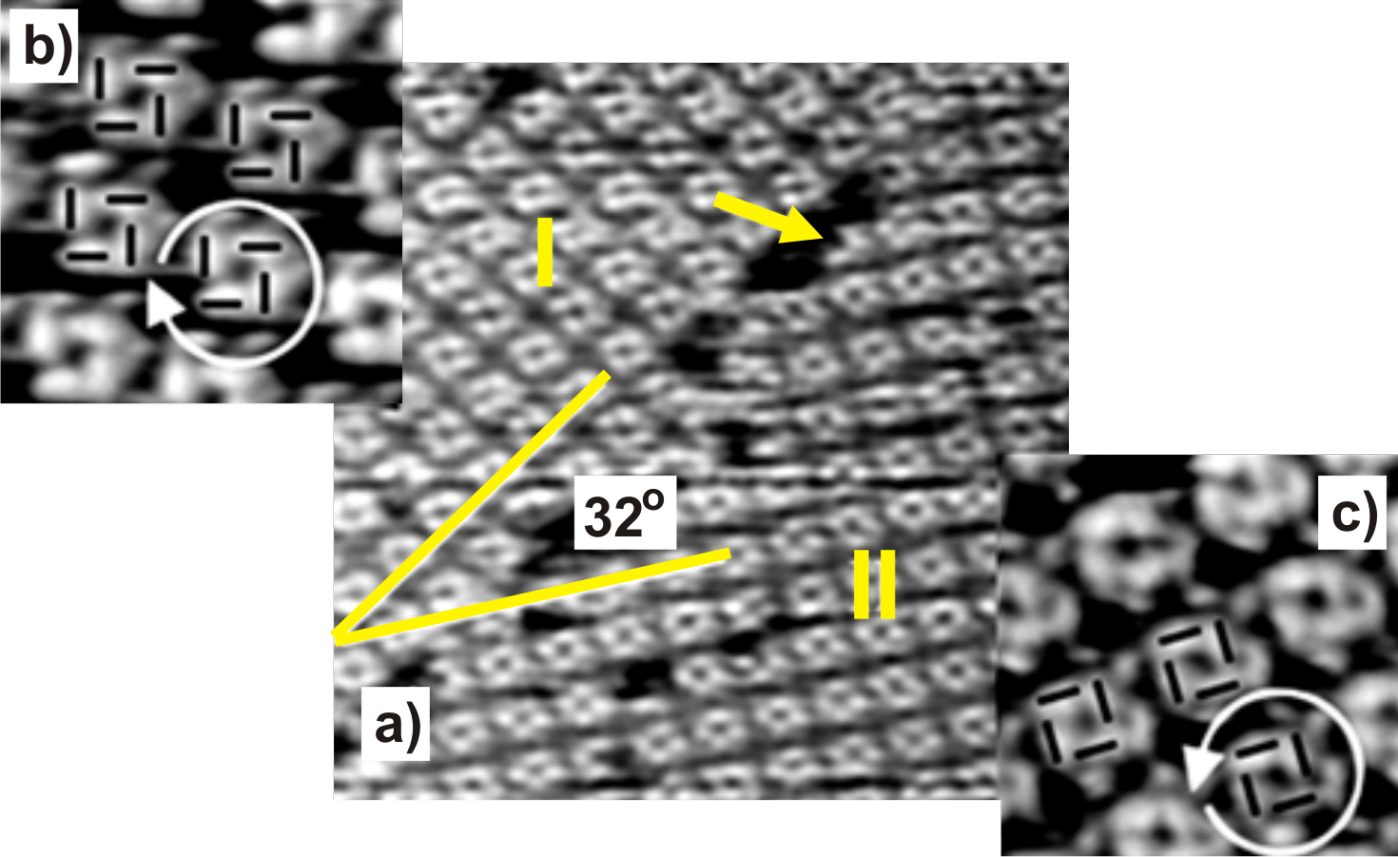
STM images of the DBV^2+^ cavitand phase on c(2 × 2)Cl/Cu(100); a) 29.2 nm × 29.2 nm, b) and c) 7.5 nm × 7.5 nm, *I*_t_ = 0.35 nA, *U*_b_ = 120 mV. Reproduced with permission from [7]. Copyright 2004 American Chemical Society.

The principles of structure formation of the adsorbed DBV species become particularly clear when comparing the findings for the ordered phases of DBV on Cl/Cu(111) with those obtained on Cl/Cu(100). To this end we summarize here very briefly the previously published results for the DBV adsorption on Cl/Cu(100) in 10 mM HCl solution [5–7]. First of all the chloride anions are known to form a well-ordered c(2 × 2) structure on the Cu(100) surface (see Figure 11) between −300 mV (near the HER) and the onset of the CDR around +150 mV vs RHE. As illustrated in Figure 9 a highly ordered layer of DBV^2+^ is observed at +50 mV on the chloride covered Cu(100) electrode in contact with the 0.1 mM DBVCl_2_ containing 10 mM HCl electrolyte, forming a so-called “cavitand”-structure consisting of small squares with a hole in the center. Figure 9a shows two mirror domains of this phase denoted as I and II enclosing an angle of 32° between them. Of course, due to the four-fold symmetry of the Cu(100) surface there exist also mirror domains I' and II' rotated by 90° resulting in four possible domains in total.

**Figure 10:**
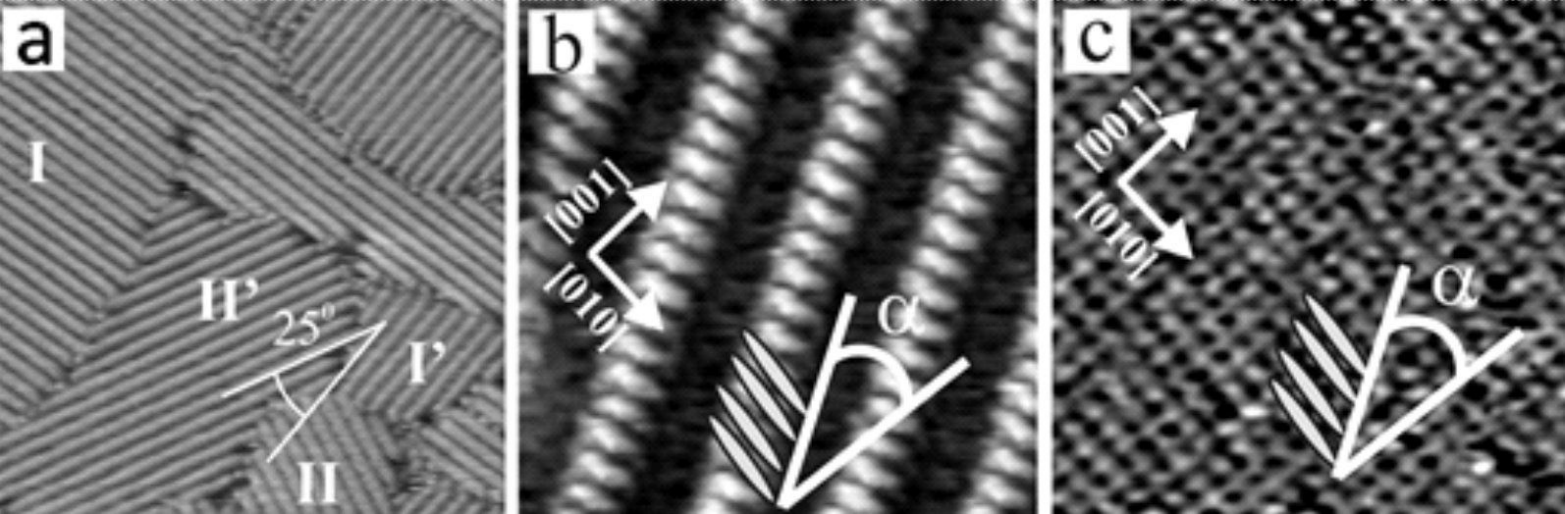
Typical STM image showing two rotational domains of the DBV^+•^ stripe pattern on Cl/Cu(100): a) 57.6 nm × 57.6 nm, *U*_b_ = 268 mV, *I*_t_ = 0.2 nA, *E* = −380 mV. Structural correlation between the stripe pattern and the underlying chloride lattice: 6.8 nm × 6.8 nm; b) *U*_b_ = 28 mV, *I*_t_ = 4 nA, *E* = −200 mV; c) *U*_b_ = 1 mV, *I*_t_ = 9 nA, *E* = −130 mV. Figure 10b and c are reproduced with permission from [5]. Copyright 2006 Royal Society of Chemistry.

A close inspection of the domain boundary in Figure 9a reveals that occasionally the small squares are incomplete (white arrow in Figure 9a) suggesting that the cavitands are actually formed from subunits. This is verified by the two high-resolution zoom-ins in Figure 9b and c. Each cavitand consists of four individual DBV^2+^ species building a square-shaped motif with a cavity in the center. Since the four building blocks may be arranged in two different ways as illustrated in the two zoom-ins these cavitands occur in two circularly chiral enantiomers [5–7]. However, since neither the Cl/Cu(100) surface nor the DBV^2+^ species (Figure 2) are chiral in nature the DBV^2+^ covered Cl/Cu(100) surface as a whole is a racemate of enantiomeric domains like I and II in Figure 9a. The dicationic character of the DBV^2+^ building blocks of this cavitand structure was verified by ex situ XPS measurements using synchrotron radiation, namely by a dominant N(1s) signal at 402.1 eV [27]. Sweeping the electrode potential to a value below −200 mV vs RHE causes the disintegration of the cavitand structure and the formation of a stripe pattern (Figure 10) similar to the one shown in Figure 7a (inset) except that here the orientation of the individual molecular species in adjacent stripes is the same and not alternating. This new phase is a consequence of the reduction of the adsorbed DBV^2+^ dications (i.e., the building blocks of the cavitand structure) to monocation radicals DBV^+•^ [5–6], which form the polymeric stripes. The distance between adjacent stripes is *s*_s_ = 1.8 nm and the intermolecular distance within the DBV^+•^ stripes is *d*_s_ = 3.6 Å. A correlation of the molecular structure of the stripes with that of the Cl-lattice underneath as deduced from STM images taken at different tunneling conditions as described above (Figure 10b,c) proves that the bipyridinium cores of the DBV^+•^species are again oriented parallel to the close-packed chloride rows underneath. Unlike on Cl/Cu(111), however, not only have molecules in adjacent rows the same orientation but all molecular stripes on the Cl/Cu(100) appear also with the same brightness (Figure 10b).

**Figure 11:**
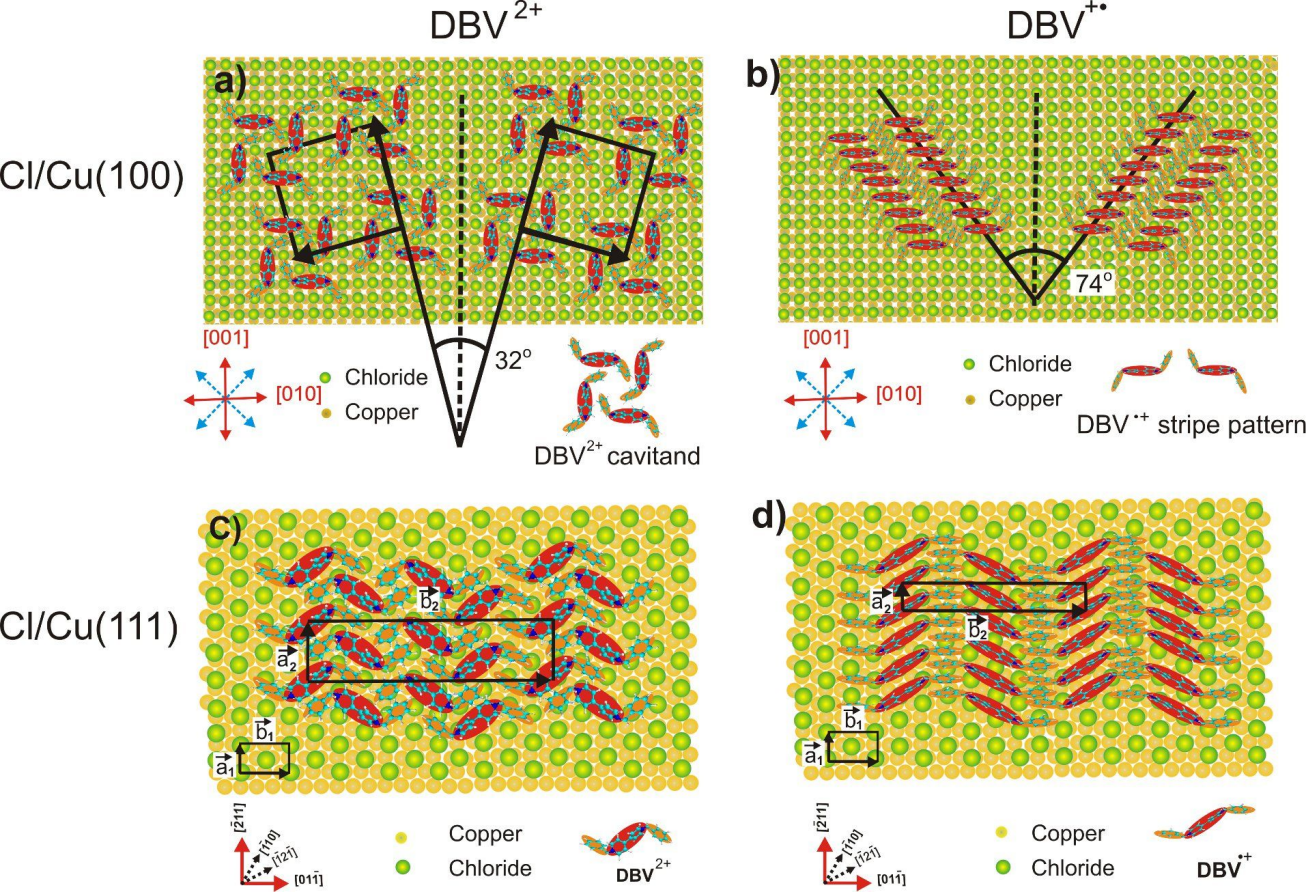
Structure models for the observed ordered layers of DBV^2+^ dications and DBV^+•^ monocation radicals on a c(p × √3)Cl/Cu(111) and a c(2 × 2)Cl/Cu(100) electrode surface, respectively, as derived from in situ STM measurements.

All observations made for the adsorption of DBV on the chloride precovered Cu(111) and Cu(100) electrode surfaces for both the dicationic DBV^2+^ and the monocation radical DBV^+•^ species, respectively, are summarized in the structural models of Figure 11a–d.

First of all in both the DBV^2+^dication and the DBV^+•^monocation radical species the positive charge resides on the N-containing bipyridinium core as revealed by the N(1s) photoemission spectra [27], while the two benzyl groups, decoupled from the delocalized π-system of the bipyridinium core by the two CH_2_ groups, are relatively more negatively charged. As a consequence the preferred adsorbate–adsorbate interactions are electrostatic attractions between the positive bipyridinium cores and the benzyl groups. In addition π–π-interactions between all π-systems of adjacent molecules will play a role with the stipulation that the benzyl groups take up a trans-conformation [6] as shown in Figure 2 and Figure 8b and in the structural models in Figure 11. In particular, the π–π interactions between neighboring monocation radicals are important which, by spin-pairing, are known to lead to the formation of dimers in solution [5]. The adsorbate–substrate interactions are obviously dominated by electrostatic interactions between the doubly (DBV^2+^) or singly (DBV^+•^) charged bipyridinium cations and the negatively charged chloride layer underneath, with the additional remark that the anion density, and thus the negative charge density, on the chloride precovered Cu(111) surface is higher than on Cl/Cu(100). This manifests itself in the fact, that the respective coverages are consistently higher on the Cl/Cu(111) surface compared to Cl/Cu(100), namely Θ(DBV^2+^) = 0.075 and Θ(DBV^+•^) = 0.20 on Cl/Cu(100) vs Θ(DBV^2+^) = 0.25 and Θ(DBV^+•^) = 0.25 on Cl/Cu(111).

Based on these possible interactions all structures of both the DBV^2+^ dications and the DBV^+•^ monocation radicals are consistently explainable (Figure 11). In all cases the positively charged bipyridinium cores are oriented parallel to the close-packed rows of the chloride anion lattice underneath in order to maximize the electrostatic attraction. This explains the parallel and 90° vs parallel and 120° orientation of both the individual DBV^2+^ or DBV^+•^ bipyridinium moieties as well as corresponding structural domains with respect to each other, on the Cl/Cu(100) and Cl/Cu(111) surface, respectively. Electrostatic repulsion between parallel oriented DBV^2+^ units leads to relatively large distances between them within the cavitand structure on Cu(100) and the “herring-bone” structure on Cu(111). Interestingly, these distances are multiples of the nearest-neighbor Cl–Cl distance, the shortest being 0.83 nm within the “herring-bone” structure on Cl/Cu(111). The directions of molecular rows of both the "alternating stripe" structure of DBV^2+^dications and the stripe phase of DBV^+•^ monocation radicals on Cl/Cu(111) are also aligned with the directions of close-packed chloride ions underneath, resulting in three possible domains in total rotated by 120° with respect to each other. The existence of the mirror domains of the cavitand structure ±16° off the direction of the two orthogonal directions of densely packed chloride rows on Cu(100) (Figure 11a), in turn, leads to four possible domains of the DBV^2+^cavitand structure on Cl/Cu(100). Likewise, the molecular rows of the DBV^+•^ stripe phase on Cl/Cu(100) propagate 37° off the direction of closely packed chloride anions. This, together with the twofold symmetry of the Cu(100) substrate, results again in a total of four possible domains of this structure on Cl/Cu(100). Summarizing so far, the occurrence of the angles of 90° and 120° and the coincidence of the connecting line between the two N-atoms of a bipyridinium core with the direction of close-packed anion rows of c(p × √3)Cl/Cu(111) and c(2 × 2)Cl/Cu(100) reflects the influence of the symmetry of the respective substrate and the dominance of electrostatic adsorbate–substrate interactions.

Looking into the fine structure of the various phases, we find the largest distance as well as an orthogonal orientation between the DBV^2+^ as a consequence of the electrostatic repulsion between these dications in the cavitand phase on Cl/Cu(100). The benzyl groups inside the cavity may partly shield this repulsion between the DBV^2+^ units (Figure 11a). Within the DBV^+•^ stripes on Cl/Cu(100) the distance between the molecular units is only 0.36 nm due to a reduced electrostatic repulsion and an attractive π–π- and spin-pairing interaction between the parallel oriented monocation radicals (Figure 11b). Similar intermolecular distances have also been observed for π–π stacked phases of 2,2'-bipyridine on Au(111) [28] and Cu(111) [26]. Even though this distance is typical for π–π-interacting aromats [29], the measured distance of 0.36 nm agrees perfectly with the separation between parallel densely packed rows of chloride anions, again a manifestation of the strong electrostatic interaction with the substrate. The lateral displacement between adjacent DBV^+•^ bipyridinium cores within the rows, leading to the mirror domains off by ±37° from the 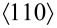 directions of the substrate, is probably due to steric hindrance between the trans-oriented benzyl groups of the DBV^+•^ units.

Also on the Cl/Cu(111) substrate the intermolecular distance of the parallel DBV^2+^ dications within the rows is large, and with 0.83 nm nearly twice the Cl–Cl distance along the commensurate direction of the c(p × √3)Cl layer between closely packed rows of the anion underlayer. This distance leaves enough space between two positively charged bipyridinium cores for a benzyl group of the neighboring row, thereby shielding the electrostatic repulsion between the former (Figure 11c). As a further consequence, the trans-conformation of the DBV^2+^ species matches the 120° orientation of adjacent bipyridinium cores within the “herring-bone” structure. The remaining benzyl groups may also π–π interact and are located within the dark lines between the “herring-bones”. The model in Figure 11d summarizes the "alternating stripe" structure formed by the DBV^+•^ species on Cl/Cu(111). Both the individual monocation radicals as well as the stripe propagation direction are aligned in 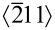 directions, molecules in adjacent rows being rotated by 120°. The intermolecular distance of 0.43 nm within the rows, though still consistent with π–π interaction, equals precisely the Cl–Cl distance in the 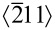 direction of the substrate, again pointing to a dominance of adsorbate–substrate interactions.

## Conclusion

The electrochemical behavior and a related structural transition of the 1,1’-dibenzyl-4,4’-bipyridinium molecule cations on a chloride-modified Cu(111) surface have been investigated by means of cyclic voltammetry and in situ scanning tunneling microscopy. Current waves in the cyclic voltammogram clearly indicate the potentials where reduction of the dications occurs first to the monocation radicals and then to the neutral molecules. At positive electrode potentials the dicationic DBV^2+^ molecules form a condensed and highly ordered "herring-bone" phase consisting of structural elements each formed by two individual DBV^2+^ molecules. In contrast, an "alternating stripe" pattern is observed for the molecules in their monocation radical form (DBV^+•^) at negative potentials below the first reduction peak. In both cases, their structural motifs are predominantly governed by dominant electrostatic interactions between the adsorbate species, both in their dication and monocation radical form, and the negatively charged chloride lattice underneath. The phase transition from the DBV^2+^-related "herring-bone" phase to the alterating stripe pattern based on the radical monocationic DBV^+•^ is observed as a reversible process occurring via nucleation and growth. Possible models for both the herring-bone phase and the alternating stripe pattern are proposed. Their detailed discussion also in the light of the corresponding findings for the same species on a c(2 × 2)Cl/Cu(100) electrode surface clearly points to a dominance of electrostatic adsorbate–substrate interactions, i.e., a strong template effect of both substrates on the self-organization of these organic surface films.

## Experimental

In this work we have employed a combination of cyclic voltammetry (CV) and electrochemical scanning tunneling microscopy (EC-STM). The direct combination of in situ STM and CV in one and the same electrochemical cell permits a precise correlation of the obtained STM images with features in the corresponding CV data. The whole experimental setup is home-built and described in detail in [25]. The tunneling tips used in all experiments were electrochemically etched from 0.25 mm in diameter tungsten wire in 2 mM KOH solution, rinsed with high purity water, dried and subsequently insulated by passing them through a hot melt glue film (ethylen–vinylacetat copolymer).

The Cu(111) single crystal used was manufactured by MaTeck, Jülich, Germany. Prior to each series of STM measurements the copper sample was electropolished by immersing it into 50% orthophosphoric acid at an anodic potential of 2 V for about 20–40 s. This removes the native surface oxide film formed in air. In order to guarantee a reproducibly smooth surface even after several electropolishing procedures, a precision of the surface orientation of less than 0.5° off the (111) plane is required. Less well defined surfaces suffer a growing roughening with repeated cycles of electropolishing.

The chloride-modified Cu(111) surface, chosen here as substrate for the viologen films, was prepared and characterized by first carrying out CV and STM measurements several times in pure 10 mM HCl solution, i.e., the supporting electrolyte. This procedure also improves the quality of the Cu(111) surface due to the operation of the so-called “electrochemical annealing effect” [17,30]. For the adsorption of the molecular film on top of the chloride-terminated electrode surface, the pure supporting electrolyte was routinely substituted by a solution of 0.1 mM 1,1’-dibenzyl-4,4’-bipyridinium dichloride in 10 mM HCl (10 mM HCl + 0.1 mM DBV^2^**^+^)** at potentials between −50 mV and +50 mV vs RHE (reversible hydrogen electrode). In this paper all potentials of the Cu(111) electrode, i.e., the working electrode, are quoted with respect to a reversible hydrogen electrode, while a Pt wire is employed as counter electrode.

High purity water from a Milli-Q purification system (conductivity > 18 MΩ·cm, TOC < 4 ppb) and highest reagent grade chemicals were used for the preparation of all solutions. All electrolyte solutions were purged with oxygen-free argon gas for several hours before use.
